# Adora2b Adenosine Receptor Engagement Enhances Regulatory T Cell Abundance during Endotoxin-Induced Pulmonary Inflammation

**DOI:** 10.1371/journal.pone.0032416

**Published:** 2012-02-28

**Authors:** Heidi Ehrentraut, Joseph A. Westrich, Holger K. Eltzschig, Eric T. Clambey

**Affiliations:** Mucosal Inflammation Program, Department of Anesthesiology, University of Colorado Denver, Aurora, Colorado, United States of America; University of Colorado Denver, United States of America

## Abstract

Anti-inflammatory signals play an essential role in constraining the magnitude of an inflammatory response. Extracellular adenosine is a critical tissue-protective factor, limiting the extent of inflammation. Given the potent anti-inflammatory effects of extracellular adenosine, we sought to investigate how extracellular adenosine regulates T cell activation and differentiation. Adenosine receptor activation by a pan adenosine-receptor agonist enhanced the abundance of murine regulatory T cells (Tregs), a cell type critical in constraining inflammation. Gene expression studies in both naïve CD4 T cells and Tregs revealed that these cells expressed multiple adenosine receptors. Based on recent studies implicating the Adora2b in endogenous anti-inflammatory responses during acute inflammation, we used a pharmacologic approach to specifically activate Adora2b. Indeed, these studies revealed robust enhancement of Treg differentiation in wild-type mice, but not in *Adora2b* ^−/−^ T cells. Finally, when we subjected *Adora2b*-deficient mice to endotoxin-induced pulmonary inflammation, we found that these mice experienced more severe inflammation, characterized by increased cell recruitment and increased fluid leakage into the airways. Notably, *Adora2b*-deficient mice failed to induce Tregs after endotoxin-induced inflammation and instead had an enhanced recruitment of pro-inflammatory effector T cells. In total, these data indicate that the Adora2b adenosine receptor serves a potent anti-inflammatory role, functioning at least in part through the enhancement of Tregs, to limit inflammation.

## Introduction

Inflammation in response to tissue injury is a carefully orchestrated process, and insufficient or overexuberant inflammation can have catastrophic effects. One major pathway by which inflammation and tissue homeostasis is regulated is through the generation of extracellular adenosine, which can serve as a highly effective “safety” signal [1]. In healthy individuals, extracellular adenosine levels are low. During tissue injury and inflammation, however, extracellular adenosine levels significantly increase due to: 1) ATP release from activated and dead/dying cells, followed by 2) generation of adenosine from ATP, ADP, and AMP, a process critically dependent on the enzyme CD73 [2], [3]. The appropriate generation and clearance of extracellular adenosine are critical in limiting tissue pathology, with mice genetically deficient either in the generation (CD73-deficient mice) or clearance (adenosine deaminase, ADA-deficient mice) of extracellular adenosine displaying profound pathologies due to inappropriate control of inflammation [3], [4], [5], [6].

Extracellular adenosine mediates its biological effects through four adenosine receptors (Adora) (Adora1, Adora2a, Adora2b, Adora3), each with distinct expression patterns and different genetic roles in regulating physiology and inflammation [7]. Based on genetic studies, adenosine-dependent signaling can play a critical role in limiting tissue damage [8], [9], [10], [11]. Significantly, the anti-inflammatory adenosine pathway is coordinately induced following injury/inflammation, with both CD73 (the extracellular adenosine-generating enzyme) and adenosine receptors (Adora2a, Adora2b) simultaneously induced [12]. Furthermore, by screening for the relative activities of multiple Adoras, studies have revealed that Adora2b (also known as the A2B receptor) is a potent anti-inflammatory receptor [9], [10], [11], [13], [14].

T cells are a major cell type of the adaptive immune system that orchestrate the immune response. Within the T cell compartment, regulatory T cells (Tregs) serve a critical function in restraining inappropriate immune responses and inhibiting inflammation. Tregs mediate these inhibitory effects by multiple mechanisms depending on the tissue and nature of injury/inflammation (e.g. production of the anti-inflammatory cytokines interleukin (IL)-10, IL-35 and transforming growth factor (TGF)-β, generation of extracellular adenosine, and cell-contact dependent mechanisms) [15], [16], [17]. Notably, Tregs can limit immune responses to a specific infection or insult (this specificity resulting from the unique T cell receptor expressed by the Tregs in question) or Tregs can have a more general, nonspecific inhibitory effect [18], [19]. While Tregs can achieve their fate either during development (natural Tregs, nTregs) or they can differentiate from a naïve T cell into an induced Treg (iTreg) [20], both cells express the transcription factor FoxP3, a critical molecular determinant essential for Treg function [21]. Recent studies have shown that extracellular adenosine is one factor that can enhance the differentiation and function of Tregs, potentially through signaling through Adora2a [22], [23], [24], [25].

While many studies have focused on the role of Adora2a in regulating T cell function, much less is known about the contribution of Adora2b and its effects on T cell differentiation. In this study, we directly investigated the Adora2b receptor as a regulator of Treg differentiation. These studies reveal a previously unappreciated role for Adora2b in promoting Tregs both in vitro and in vivo, and identify a previously unappreciated mechanism by which Adora2b might limit inflammation.

## Results

### A pan-adenosine receptor agonist enhances the abundance of Tregs following in vitro stimulation of primary mouse T cells

**Figure 1:**
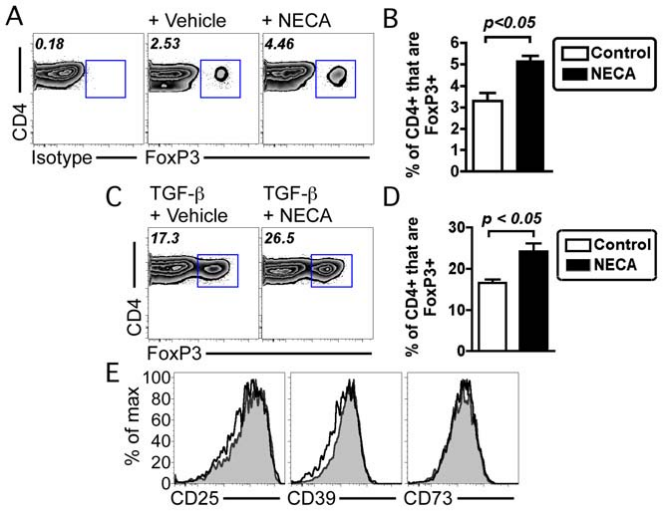
Adenosine receptor activation enhances the abundance of Tregs following in vitro stimulation of primary mouse T cells. (A) Adenosine receptor activation enhances the abundance of Tregs following antibody-mediated stimulation of T cells (using anti-CD3 antibody, 1 µg/mL combined with 10 ng/mL of IL-2, in the absence of TGF-β). Samples were either untreated (+Vehicle) or treated with 10 µM of NECA (+NECA), a potent adenosine receptor agonist, analyzed three days post-stimulation for the relative abundance of FoxP3-expressing Tregs. The numbers present in the upper left-hand corner of each flow cytometry plot indicate the percentage of cells that are FoxP3+ as defined by the square gate. Background staining with an isotype control antibody is indicated in the leftmost panel. (B) Quantitation of FoxP3-expressing cells following either control (vehicle treated, white bars) or NECA treated (black bars), with data indicating mean +/− SEM of triplicate cultures done in two separate experiments. (C) Adenosine receptor activation enhances the abundance of Tregs following antibody-mediated stimulation of T cells in the presence of TGF-β, a known inducer of Tregs (using anti-CD3 antibody, 1 µg/mL combined with 10 ng/mL of IL-2, combined with 0.75 ng/mL TGF-β), showing flow cytometric analysis (C) and quantitation (D). The numbers present in the upper left-hand corner of each flow cytometry plot indicate the percentage of cells that are FoxP3+ as defined by the square gate. Relative abundance of Tregs within cultures were defined by flow cytometry, with Tregs defined as live, MHC class II negative, CD8−, CD4+ cells that express the transcription factor FoxP3. Data indicate mean +/− SEM of triplicate cultures, representative of two independent experiments. (E) Tregs generated by TGF-β with NECA (solid black line) relative to Tregs generated by TGF-β treatment alone (indicated in gray) have a comparable cell surface expression of CD25, CD39 and CD73. Results representative of results from three independent cultures, done in two independent experiments. Statistical analysis was performed using unpaired t test, with statistically significant differences as indicated.

Given the potent anti-inflammatory effects mediated by extracellular adenosine, we tested the effect of a general adenosine receptor agonist (NECA) on the relative abundance of Tregs, an anti-inflammatory subset of T cells, after in vitro stimulation of primary murine T cells. Consistent with previous reports [24], NECA stimulation enhanced the relative abundance of FoxP3 expressing Tregs in stimulated splenocyte cultures (Fig. 1A–B).

TGF-β is a potent inducer of FoxP3 expression and an inducer of Tregs. Adenosine receptor stimulation further enhanced Tregs differentiation in the presence of TGF-β (Fig. 1C–D). Tregs present following adenosine receptor stimulation had comparable expression relative to normal Tregs based on their cell surface expression of three proteins expressed on Tregs, CD25, CD39 and CD73 (Fig. 1E), indicating that adenosine receptor stimulation did not perturb the general phenotype of Tregs after in vitro culture.

**Figure 2:**
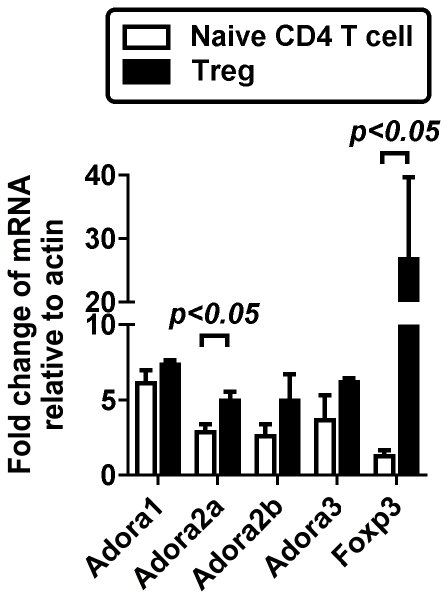
Relative expression levels of adenosine receptor genes in T cell subsets. Real-time PCR analysis of mRNA for the four adenosine receptor genes in FACS purified CD4 T cell subsets of naïve CD4 T cells or Tregs, with Tregs isolated from FoxP3GFP or DEREG mice. Values were standardized to bulk spleen mRNA, with each value showing expression relative to actin. High level expression of FoxP3 mRNA is consistent with a highly purified population of Tregs. Data representative of two to three independent experiments, analyzing at least three independently isolated populations for both naïve CD4 T cells and Tregs. Data depict mean ± SEM for each transcript. Statistically significant differences were calculated by unpaired t test comparing expression in naïve CD4 T cells relative to Tregs, as indicated.

To define potential mechanisms by which adenosine receptor engagement might enhance Treg abundance, we measured relative expression levels of the four adenosine receptors in both naïve CD4 T cells and Tregs. This analysis identified that both T cell subsets expressed all four of the adenosine receptors, with mRNA expression of Adora2a and FoxP3 upregulated in Tregs relative to naïve CD4 T cells (Fig. 2).

### An Adora2b-specific agonist enhances Treg abundance in vitro following activation of murine T cells

**Figure 3:**
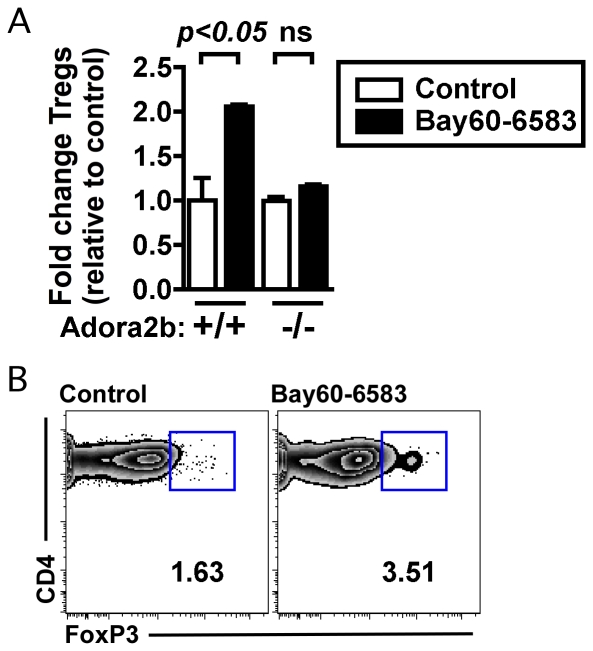
An Adora2b-specific agonist enhances Treg abundance in vitro following activation of murine T cells. Bulk splenocytes from either *Adora2b*+/+ (C57BL/6J) or *Adora2b*−/− mice were cultured with soluble anti-CD3 for three days with or without the Bay60-6583 compound, at which time the relative abundance of Tregs was assessed. (A) Fold change in Treg abundance relative to *Adora2b*+/+ cultures without the Bay compound control, where Tregs were defined as viable, CD4+ FoxP3+ cells by flow cytometry. (B) Flow cytometry plots shown from *Adora2b*+/+ cultured splenocytes. Bay60-6583, an Adora2b-specific agonist was added to a final concentration of 4 nM. Data from two independent experiments, each containing 1–3 independent replicates. The numbers present on each flow cytometry plot indicate the percentage of cells that are FoxP3+ as defined by the square gate, with FoxP3 expressing cells defined relative to isotype control-stained samples (not shown). Statistically significant differences are indicated and were calculated by one-way ANOVA followed by Bonferroni's post-test correction. ns indicates a comparison that is not statistically significantly different.

Previous studies have implicated the Adora2a adenosine receptor in promoting Treg abundance [24]. Given the expression of the Adora2b receptor in both naïve T cells and Tregs, and the accumulated evidence of an anti-inflammatory effect for Adora2b in vivo, we directly tested the consequence of an Adora2b-specific agonist (Bay60-6583) [13] on in vitro cultures of activated primary murine T cells. While Bay60-6583 treatment of control *Adora2b*+/+ splenocytes resulted in an increased frequency of Tregs after three days of culture, this compound had no effect on Treg abundance in *Adora2b*−/− cultures (Fig. 3). These data identify that an Adora2b-specific agonist is capable of enhancing Tregs in vitro.

### Adora2b-deficient mice fail to increase Tregs after endotoxin-induced pulmonary inflammation

**Figure 4:**
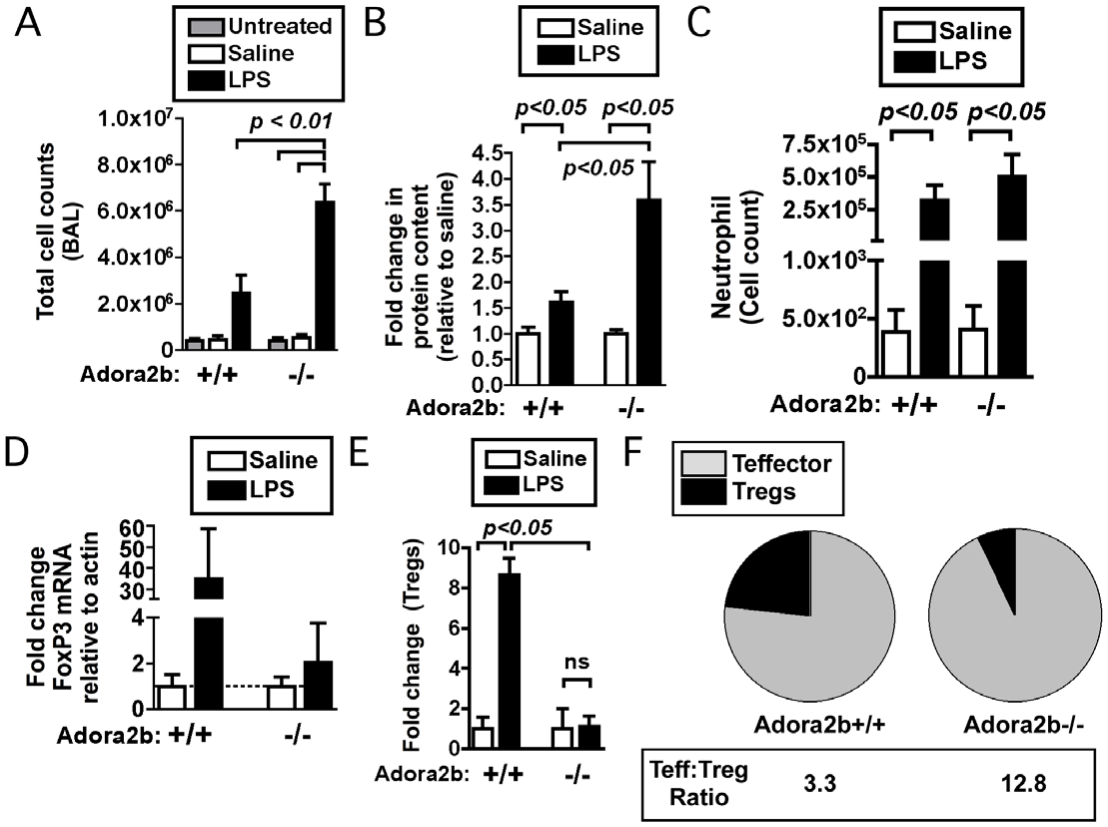
*Adora2b*-deficient mice have increased inflammation with impaired induction of Tregs after LPS-induced lung injury. LPS-induced inflammation was initiated in 9–12 wk-old male *Adora2b*+/+ (C57BL/6J) or age and weight matched *Adora2b*−/− mice by intratracheal instillation of LPS or saline. Bronchoalveolar lavage (BAL) fluids were collected 1 d post treatment. A) Leukocyte cell counts of BAL fluid were determined utilizing a cellometer (mean ± SEM, n = 2–4). B) Protein content of BAL fluid was determined with BCA assay. Fold increase of protein concentration in LPS treated BAL samples over respective saline BAL samples is displayed (mean ± SEM, n = 3–10). C) Analysis of cell infiltration into the lung airspace (BAL) of mice exposed to inhaled LPS. Mice (*Adora2b*+/+ or *Adora2b*−/−) were exposed to nebulized saline or lipopolysaccharide (LPS); 24 hours post-exposure, mice were euthanized, and BAL was harvested. Cells were then subjected to flow cytometric analysis to identify neutrophils (highly granular, Gr1+ cells). D) Analysis of FoxP3 gene expression in lungs of mice exposed to inhaled LPS. Mice (*Adora2b*+/+ or −/−) were exposed to nebulized saline or lipopolysaccharide (LPS); 24 hours post-exposure, mice were euthanized, and perfused lungs were harvested. Total RNA was harvested from lungs, cDNA prepared and analyzed by real-time PCR. Data depict mean (± SEM) fold changes in indicated genes. Fold change was calculated based on primer efficiencies, standardized to changes in actin, with *Adora2b*+/+ Saline mean value defined as 1. E) Fold change in cell number of either regulatory T cells (lymphocyte size, CD3+ CD4+ FoxP3+) in the lung airspace (BAL) of mice exposed to inhaled saline or LPS as above. Data are from 2–6 mice; 2 independent experiments. F) Relative abundance of CD4 effector T cells (Teffs, defined as lymphocyte size, CD3+ CD4+ CD44high cells) to Tregs based on cell counts from either *Adora2b*+/+ or *Adora2b*−/− mice, with pie charts depicting mean cell count for the indicated cell populations from 2–6 mice; 2 independent expts. Analysis of total cellular infiltration into airways and protein leakage (panels A–B) was done in animals treated with intratracheal LPS. Analysis of neutrophil infiltration and Tregs (panels C–F) was done in animals treated with inhaled LPS. Statistically significant differences are indicated and were calculated either by one-way ANOVA (A) or by unpaired t test (B–E) with a focus on whether *Adora2b*−/− LPS treated animals were statistically different from either saline treated or *Adora2b*+/+ LPS treated mice.

We next sought to investigate the in vivo consequence of Adora2b deficiency on T cell populations during pulmonary inflammation. Previous studies have revealed that *Adora2b*-deficient mice have enhanced pulmonary inflammation, marked by increased neutrophil infiltration, elevated levels of pro-inflammatory cytokines (e.g. IL-6, TNF-α), and decreased levels of the anti-inflammatory cytokine IL-10 [26]. Consistent with these earlier studies, we found that lipopolysaccharide (LPS) exposed *Adora2b*−/− mice had increased cellularity in the airways after LPS exposure and increased protein leakage into the airways relative to wild-type controls (Fig. 4A–B), with pronounced neutrophil infiltration (Fig. 4C). Given the ability of an Adora2b agonist to enhance Tregs, we tested the relative change in Treg abundance in *Adora2b*−/− mice following LPS exposure. Notably, *Adora2b*−/− mice failed to induce an increase in Tregs in the lung or the airway after LPS exposure (Fig. 4D, E). Conversely, *Adora2b*−/− mice had an increased infiltration of CD4 effector T cells, such that the relative ratio of CD4 effector T cells to Tregs in *Adora2b*−/− was elevated relative to *Adora2b*+/+ controls (Fig. 4F). The failure to increase Treg abundance, coupled with an enhanced recruitment of CD4 effector T cells identifies Adora2b as a critical regulator which influences the relative contribution of anti-inflammatory Tregs to pro-inflammatory effector T cells during inflammation.

## Discussion

During acute inflammatory responses, the generation of extracellular adenosine is an important feedback mechanism that limits inflammation [1], [8]. The stepwise generation of extracellular adenosine by CD39 and CD73 from ATP is thought to limit inflammation by at least two ways: i) through the enzymatic degradation of extracellular ATP, which can promote inflammation [27] and ii) by extracellular adenosine directly signaling through adenosine receptors [1]. Notably, signaling through adenosine receptors triggers multiple anti-inflammatory pathways including the induction of IL-10 [28], the impaired immunogenicity of dendritic cells [29], and the deneddylation of cullin-1 to limit NF-κB activation [30]. In this manuscript, we present data for a new anti-inflammatory mechanism downstream of Adora2b: Adora2b-dependent induction of regulatory T cells.

The potent anti-inflammatory effects of Adora2b during acute inflammation have been revealed in multiple studies of acute inflammatory insults including studies of localized or systemic microbial challenge [11], [14], [31], [32], [33]. Adora2b also has a pronounced anti-inflammatory role in the context of ischemic tissue injury (i.e. transient tissue hypoxia) such that *Adora2b*-deficient mice have more severe acute ischemic injury in studies of both renal and myocardial ischemia [9], [10]. Notably, hypoxia elicits multiple adaptive responses within cells to deal with limited oxygen availability, including induction of the extracellular adenosine sensing pathway [12], [34], [35], induction of toll-like receptors TLR2 and TLR6 [36], activation of the NF-κB machinery [37], and upregulation of integrins which modulate cell trafficking [38]. Given the integration of hypoxic sensing machinery with Adora2b, future studies will focus on how hypoxia and extracellular adenosine signaling intersect in regulating the generation of Tregs. This work is especially relevant given our recent studies demonstrating that hypoxia can enhance the generation of Tregs, thereby restricting hypoxia-associated inflammation (Clambey *et al*, submitted).

While Adora2b signaling can be tissue protective, Adora2b can also inappropriately restrict inflammation. This balance between tissue-protection versus host-defense has been nicely revealed by studies of cecal ligation and puncture, in which *Adora2b*-deficient mice were more resistant to sepsis and sepsis-associated mortality [33]. Furthermore, Belikoff *et al* found that an Adora2b antagonist was capable of increasing survival in septic mice, even those, that based on increased levels of IL-6 in the blood, were predicted to succumb to mortality [33].

In contrast to the potential beneficial effects of Adora2b in acute inflammatory contexts, this receptor can be detrimental in conditions of prolonged inflammation [39], [40], [41]. For example, in elegant studies from the Blackburn laboratory, the pulmonary inflammation and fibrosis observed in adenosine deaminase (ADA) deficient mice is significantly improved upon treatment of these mice with an Adora2b-specific antagonist [39]. Surprisingly, however, Adora2b genetic deficiency worsened ADA-deficient inflammation [42]. This apparent discrepancy in the role of Adora2b is likely due to the kinetics of Adora2b loss-of-function, where pharmacological studies focused on the effects of blocking Adora2b after the onset of inflammation [39] in contrast to genetic ablation of Adora2b that occurred prior to the induction of inflammation [42]. Consistent with this idea, direct comparison of acute and chronic models of bleomycin-induced lung injury demonstrated that while Adora2b served a potent anti-inflammatory role during acute lung injury, Adora2b had little effect on inflammation and was instead pro-fibrotic during chronic pulmonary fibrosis [43]. The pathogenic potential of Adora2b in chronic inflammation is not restricted to the lung. For example, Adora2b signaling was recently revealed to be detrimental in sickle cell anemia, a context in which elevated levels of extracellular adenosine-Adora2b signaling promotes red blood cell sickling, contributing to the pathogenesis of this disease [44].

Based on our current observations that Adora2b enhances Tregs, it is interesting to speculate that some of the detrimental effects of Adora2b in chronic pathologies may be due to excessive generation or function of Tregs. A detrimental role for an adenosine-driven Treg pathway may be particularly relevant in the context of elevated extracellular adenosine levels (e.g. in pulmonary fibrosis, sickle cell anemia, fibrosis or solid tumors [5], [44], [45], [46]). In fact, recent data indicate that Tregs may participate in the process of fibrosis [47], [48], with a pro-fibrotic outcome occurring through increased Treg production of TGF-β1 and subsequent collagen production following immune activation [49].

The divergent effects of Adora2b in acute and chronic inflammatory contexts indicate that Adora2b function is likely to be shaped by the cells and environments in which inflammation is occurring. Our data define a role for Adora2b in enhancing Tregs either in primary activated murine T cell cultures or after LPS exposure, a finding consistent with a recent report showing that antagonizing Adora2b signaling inhibits the generation of Tregs in vitro [50]. In contrast to our findings, however, a recent paper reported that Adora2b promoted the generation of pro-inflammatory Th17 cells [51]. While the explanation for this apparent discrepancy remains to be elucidated, it is notable that the Th17-promoting effects of Adora2b in these studies were isolated to effects of Adora2b specifically on dendritic cells, and not on macrophages [51]. This observation raises the possibility that the contribution of Adora2b to T cell differentiation depends on the type of antigen presenting cell (e.g. dendritic cell versus macrophage) and microenvironment. For example, while treatment of dendritic cells with NECA induces IL-6 expression in an Adora2b-dependent mechanism [51], *Adora2b*-deficient mice or macrophages had increased levels of IL-6 during acute inflammation, indicating that Adora2b can limit IL-6 in certain contexts [26], [33]. This cell-type specific regulation of IL-6 by Adora2b is particularly relevant to understanding how Adora2b could either induce anti-inflammatory Tregs, as we show here, or pro-inflammatory Th17 cells [51], given that Treg differentiation can be diverted to Th17 differentiation in the presence of IL-6 [52]. It will be important for future studies to elucidate the cell-type specific contributions of Adora2b in controlling both acute and chronic inflammatory responses.

The central role of Adora2b in determining the outcome of both acute and chronic pathologies identifies this molecule as a promising point of intervention. Since Adora2b serves as a receptor both for extracellular adenosine as well as for alternative ligands (e.g. Netrin-1, [53], [54], [55]), this receptor may function to integrate multiple extrinsic cues to promote Tregs to restrict the duration and magnitude of inflammation. Pharmacologic targeting of adenosine pathways such as Adora2b may also synergize with modalities that activate the hypoxic response and have been shown to be tissue-protective in models of colitis [56], [57]. Conversely, since both Adora2a and Adora2b signaling promote Tregs, transient depletion of extracellular adenosine through the administration of pegylated-ADA may be a potent method to transiently limit the generation or activity of Tregs. Based on the potential of Adora2b-targeted treatments to modulate regulatory T cells, future studies will interrogate the consequences and therapeutic benefits of manipulating the Adora2b-Treg axis in both acute and chronic states of inflammation.

## Methods

### Mice

*Adora2b*+/+ (C57BL/6J) and *Adora2b*−/− [13] mice were bred in house. B6.Cg-*Foxp3^tm2Tch^*/J (here referred to as FoxP3GFP [58]) were obtained from Jax and DEREG mice were kindly provided by Dr. Tim Sparwasser [59]. All mouse experiments were done using age- and sex-matched mice, with mice typically used between 8–12 weeks of age. The animal protocol was approved by the Institutional Animal Care and Use Committee of the University of Colorado Denver (under Animal Welfare Assurance Policy A3269-01, IACUC protocol B83708(07)1D) and is in accordance with the National Institutes of Health guidelines for use of live animals. The University of Colorado Denver, Anschutz Medical Campus is accredited by the American Association for Accreditation of Laboratory Animal Care (#00235).

### Tissue harvest

At time of harvest, bronchoalveolar lavage (BAL) was harvested by performing three sequential lavages of the airways, using an ice-cold PBS solution containing 5 mM EDTA. Lungs were harvested from mice, following perfusion of the lungs using saline. For lungs subjected to flow cytometric analysis, lung tissue was mechanically disrupted by scissors, followed by a one-hour incubation of lung tissue with collagenase D at a final concentration of 1 mg/mL at 37 C. Following collagenase treatment, lung material was placed over a 100 micron mesh and cells were forced through using the plunger of a 3 mL syringe and multiple washes of disrupted material.

### T cell purification

T cells were purified from the spleen of mice, mechanically disrupted over a 100 micron filter, subjected to magnetic bead enrichment for CD4 T cells using a CD4 T cell isolation kit (Miltenyi Biotec, Germany). Enriched CD4 T cells were then subjected to FACS purification on a FACSAria (BD), with naïve CD4 T cells purified based on the cell surface phenotype CD4+ CD62L^high^ CD44^low^. FoxP3-expressing Tregs were purified from two different genetically engineered mice which express GFP specifically in Tregs, the FoxP3GFP (B6.Cg-*Foxp3^tm2Tch^*/J from Jax [58], [60]) and DEREG mice (kindly provided by Dr. Tim Sparwasser [59]). In both strains of mice, GFP expression is directly linked to the regulatory sequences from the FoxP3 locus, either using an internal ribosome entry site at the end of the endogenous FoxP3 gene (in FoxP3GFP mice) or using a bacterial artificial chromosome-based transgenic in which GFP was placed downstream of the FoxP3 promoter (in DEREG mice). FoxP3 expressing cells were purified for subsequent qPCR analysis, with Tregs identified and isolated by sorting as CD4+ GFP+ [58], [60].

### In vitro T cell cultures

Bulk splenocytes or purified CD4 T cells were cultured in Iscove's modified Dulbeco's medium (IMDM, Gibco) containing 5% FBS, L-glutamine, penicillin/streptomycin and β-mercaptoethanol (50 µM). All cultures were done using cells at a concentration of 1×10^6^ cells/mL. For cultures subjected to T cell receptor stimulation, bulk splenocytes were stimulated with 1 µg/mL soluble anti-CD3ε (clone 145-2C11, eBioscience), supplemented with 10 ng/mL of recombinant mouse IL-2 (eBioscience). In vitro Treg differentiation assays were done for 3 days. In certain assays, exogenous TGF-β was added to the culture, using recombinant human TGF-β1 (eBioscience). For assays in which cells were stimulated with a pan receptor agonist, cultures were treated with 10 µM NECA (5′-N-Ethylcarboxamidoadenosine, Tocris Bioscience, MO, USA) for the duration of the culture. For cultures stimulated with the Adora2b-specific agonist Bay60-6583, cultures were treated with 4 nM Bay60-6583 (obtained from Bayer, Germany).

### RNA isolation and Real-time PCR

Total RNA was extracted from cells or tissue by Trizol, followed by cDNA synthesis using iScript cDNA Synthesis Kit (Bio-Rad Laboratories, Inc, Munich, Germany) according to the manufacturer's instructions. Quantitative real-time reverse transcriptase PCR (qPCR) (iCycler; Bio-Rad Laboratories, Inc.) was performed to measure relative mRNA levels for various transcripts, with qPCR master mix contained 1 µM sense and 1 µM antisense primers with iQ™ SYBR® Green (Bio-Rad Laboratories, Inc.). For every assay, melt curve analysis was performed and samples with aberrant melt curves were discarded. All qPCR assays were standardized relative to β-actin levels.

### Antibodies and Flow cytometric analysis

Antibodies were purchased from eBioscience unless otherwise noted. Anti-mouse antibodies included CD4 (GK1.5), CD25 (PC61.5), CD39 (24DMS1), CD73 (eBioTY/11.8 (TY/11.8)), FoxP3 (FJK-16s), Gr1 (RB6-8C5), and MHC class II (M5/114.15.2). Single cell suspensions of cultured cells or disrupted tissue were stained with a cocktail of antibodies for 30 minutes at room temperature in the dark, in staining buffer containing an anti-Fc receptor antibody (2.4G2). When cells were stained for FoxP3, staining was done using FoxP3 staining buffer according to manufacturer's instructions (eBioscience). Unless stated otherwise, all stains included a viability dye to identify viable cells (LIVE/DEAD® Fixable Aqua Dead Cell Stain Kit, Invitrogen), with Tregs routinely identified as lymphocytes (based on forward and side scatter profiles) that were viable, MHC class II negative cells, which expressed CD4 and FoxP3. FoxP3-expressing Tregs were identified by comparing fluorescent signal between samples stained with an isotype control antibody (background staining), relative to samples stained with a FoxP3-specific antibody. Isotype stained controls routinely had less than 0.5% positive events within the FoxP3+ gate. All flow cytometry was done on an LSRII (BD), with compensation done using FACSDiva software. All flow cytometry data is show on a log_10_ scale.

### Primers for qPCR analysis

All primers used for qPCR are indicated below, with a 5′ to 3′ orientation, and m referring to mouse. mbeta-actin: Fwd-CTAGGCACCAGGGTGTGAT, Rev-TGCCAGATCTTCTCCATGTC; mFoxP3: Fwd-TCTCCAGGTTGCTCAAAGTC, Rev-GCAGAAGTTGCTGCTTTAGG; mAdora1: Fwd-AGAACCACCTCCACCCTTCT, Rev-TACTCTGGGTGGTGGTCACA; mAdora2a: Fwd-GAAGCAGATGGAGAGCCAAC, Rev-GAGAGGATGATGGCCAGGTA; mAdora2b: Fwd-TGCATGCCATCAACTGTATC, Rev-TGGAAACTGTAGCGGAAGTC; mAdora3: Fwd-TCCCTGATTACCACGGACTC, Rev-CAATTCGCTCCTTCTGTTCC.

### Murine LPS exposure models

Male *Adora2b*+/+ (C57BL/6J mice, The Jackson Laboratory, ME) and *Adora2b*−/− on a C57BL/6J background were randomly assigned to saline or LPS treatment groups at age 9–12 wk. LPS from *Escherichia coli* 0111:B4 (L4391, Sigma-Aldrich) was dissolved in 0.9% saline (2 mg/mL). Animals were anesthetized with pentobarbital (70 mg/kg i.p.). A volume of either 50 µL LPS (100 µg/animal) or saline was instilled intratracheally via a 22-gauge canule, followed by 0.1 mL of air. Animals were harvested 24 hours after instillation. For studies in which mice were exposed to aerosolized LPS, mice were exposed to aerosolized LPS in a cylindrical chamber connected to an air nebulizer (MicroAir; Omron Healthcare, Mannheim, Germany) as published previously [26].

### Measurement of BAL fluid protein content

After collection samples were centrifuged for 1 min at 3,000× g, and supernatant was stored at −80°C. Colorimetric Pierce bicinchoninic acid (BCA) protein assay (Thermo Scientific) was performed according to the manufacturer's protocol to determine protein content of BAL supernatant.

### Leukocyte counts of BAL fluid

BAL samples were mixed gently prior to diluting 50 µL of BAL fluid with 50 µl of Trypan blue (1∶5 in 1× PBS) for viable cell counts. 20 µl of diluted cell sample were pipetted on a cellometer cell counting chamber. Leukocytes were automatically counted using a Cellometer Auto T4 (Nexcelom Bioscience, Lawrence, MA, USA).

### Software & statistical analysis

Data analysis and plotting were done using Prism 4.0c (GraphPad Software, Inc, San Diego, CA). Flow cytometric data were analyzed using FlowJo (TreeStar, Inc, Ashland, OR), with data displayed as high-resolution zebra plots showing outliers, using log_10_ scales. Statistical analyses were performed using Prism 4.0c, with unpaired t-tests or one-way ANOVA and Bonferroni's multiple comparison post-test correction for all other analyses.
